# Slack K^+^ channels limit kainic acid-induced seizure severity in mice by modulating neuronal excitability and firing

**DOI:** 10.1038/s42003-023-05387-9

**Published:** 2023-10-11

**Authors:** David Skrabak, Helmut Bischof, Thomas Pham, Peter Ruth, Rebekka Ehinger, Lucas Matt, Robert Lukowski

**Affiliations:** Department of Pharmacology, Toxicology and Clinical Pharmacy, Institute of Pharmacy, University of Tübingen, Tübingen, Germany

**Keywords:** Epilepsy, Ion channels in the nervous system

## Abstract

Mutations of the Na^+^-activated K^+^ channel Slack (*KCNT1*) are associated with terrible epilepsy syndromes that already begin in infancy. Here we report increased severity of acute kainic acid-induced seizures in adult and juvenile Slack knockout mice (Slack^−/−^) in vivo. Fittingly, we find exacerbation of cell death following kainic acid exposure in organotypic hippocampal slices as well as dissociated hippocampal cultures from Slack^−/−^ in vitro. Furthermore, in cultured Slack^−/−^ neurons, kainic acid-triggered Ca^2+^ influx and K^+^ efflux as well as depolarization-induced tetrodotoxin-sensitive inward currents are higher compared to the respective controls. This apparent changes in ion homeostasis could possibly explain altered action potential kinetics of Slack^−/−^ neurons: steeper rise slope, decreased threshold, and duration of afterhyperpolarization, which ultimately lead to higher action potential frequencies during kainic acid application or injection of depolarizing currents. Based on our data, we propose Slack as crucial gatekeeper of neuronal excitability to acutely limit seizure severity.

## Abstract (web summary)

Mice lacking the Na ^+^ -activated K^+^ channel, Slack, exhibit increased severity of acute kainic-acid induced seizures, suggesting a crucial role for this channel in neuronal excitability.

## Introduction

The sodium ion (Na^+^)-activated potassium ion (K^+^) channel Slack (sequence like a calcium ion (Ca^2+^)-activated K^+^ channel, K_Na_1.1, Slo2.2) is predominantly expressed in central and peripheral neurons^1–4^. Due to its high conductance, Slack effectively regulates neuronal excitability by limiting action potential (AP) frequency^5–8^, increasing afterhyperpolarization (aHP)^9,10^, and setting the resting membrane potential (RMP)^11–14^. As Slack channels are gated by high intracellular Na^+^ concentrations ([Na^+^]_i_) of ~40 mmol/l^15,16^, they are thought to activate under physiological conditions by forming functional microdomains with Na^+^ sources. Respective physical or functional interactions of Slack were demonstrated with voltage-activated Na^+^ channels^17^, α-amino-3-hydroxy-5-methyl-4-isoxazole propionic acid (AMPA)- ^18–20^ and N-methyl-D-aspartate (NMDA)-type glutamate receptors^21,22^.

Mutations of the Slack gene *KCNT1* are linked to at least two devastating childhood epilepsy syndromes that are, so far, mostly intractable by common antiepileptic drugs^23,24^. *Epilepsy of Infancy with Migrating Focal Seizures* (EIMFS) shows onset in the first weeks of life leading to severe developmental delay^25^ while *Autosomal Dominant Nocturnal Frontal Lobe Epilepsy* (ADNFLE) is a serious non-rapid eye movement (REM) sleep-related frontal lobe epilepsy with onset at an age of approximately 5 years^24–27^. Currently described disease-causing mutations are predominantly gain of function (GOF) mutations, that increase K^+^ conductance, open probability, Na^+^ sensitivity, or inter-subunit cooperativity, with the latter resulting in intermediate conductance states^28–30^. Interestingly, also a loss of function (LOF) variant of Slack was described to cause severe early infantile epilepsy^31,32^, while increased susceptibility to glutamate excitotoxicity was recently reported for global Slack^−/−^ mice^21^. These facts suggest a central role of Slack in the control of neuronal excitability, as both, too much as well as too little Slack activity increases seizure susceptibility.

In this study, using an in vivo epilepsy model, neuronal culture systems, live-cell imaging of Ca^2+^ and K^+^, and electrophysiology, we identify a neuroprotective role of Slack channels during acute epilepsy.

## Methods

### Animals

All experimental procedures were conducted in accordance with animal protection law in Germany and approved by the Ethics Committee for Animal Research (Regierungspräsidium Tübingen). Animals were maintained on a 12/12 h light/dark cycle (light on 6 a.m. to 6 p.m.) with access to food and water ad libitum. Slack-deficient mice (B6.129-Kcnt1^tm1Ruth^/RoLu) were generated by targeted ablation of the Slack encoding gene *Kcnt1* in murine embryonic stem cells using a Cre/loxP-system^33^. Founders were backcrossed to C57BL/6 N background for at least nine generations. Genotypes were determined by PCR after DNA extraction by KAPA2G Fast HotStart Genotyping Mix (KK5621, KAPA Biosystems, Sigma Aldrich Chemie GmbH, Taufkirchen, Germany). Primer sequences are listed in Supplementary Table 1. KA-based epilepsy model was performed with either 4 weeks-old (juvenile) or 12 weeks-old (adult) male litter-matched offspring from heterozygous (Slack^+/−^) parental (P_2_) animals. Subsequently, homozygous Slack^+/+^ or Slack^−/−^ animals (P_1_) not used in epilepsy experiments were mated to generate age-matched homozygous Slack^+/+^ or Slack^−/−^ pups for in vitro experiments i.e., hippocampal brain slice cultures or dissociated cell cultures. This two-stage breeding strategy avoids the need to genotype P0 to P1 pups used for culturing, as would be required when using heterozygous breeders. At the same time, genetic drift between individual homozygous lines is prevented.

### Kainic acid model for acute epilepsy

Susceptibility and seizure severity in Slack^+/+^ and Slack^−/−^ mice were assessed by using the KA-based model of acute epilepsy. KA (ab120100, Abcam, Berlin, Germany) dissolved in sterile saline was intraperitoneally applied to randomly selected litter-matched juvenile (20 mg/kg) or adult (30 mg/kg) male Slack^+/+^ and Slack^−/−^. Injected animals were transferred to a vivarium for optimal observation and video recording for 90 min. Seizure severity was measured as seizure score (SSc) on an adopted Racine Scale as highest score reached in a 5 min interval. Score criteria ranged from SSc 0—no response, to SSc 1—immobile and rigid, SSc 2—myoclonic jerks and head nodding, SSc 3—forelimb clonia and Straub-tail, SSc 4—rearing with forelimb clonia, SSc 5—rearing, falling and jumping, SSc 6—tonic-clonic seizures with status epilepticus (SE) and SSc 7—seizures leading to death. Following observation, animals recovered in their home cage for 24 h before euthanasia. Hippocampi were dissected from removed brains in ice-cold DPBS (14190-094, Thermo Fisher Scientific, Waltham, US) and snap-frozen in liquid nitrogen for subsequent mRNA isolation.

### Organotypic hippocampal slice cultures

Hippocampal slice cultures (HSC) were obtained from postnatal day 5 (P5) pups of both sexes. Pups were decapitated and brains removed. Hippocampi were dissected in ice-cold dissecting medium, composed of HBSS (14025, Thermo Fisher Scientific), 20 mM HEPES, and 20 mM D-glucose (both obtained from Carl Roth GmbH + Co. KG, Karlsruhe, Germany) using an iris spatula (10093-13, Fine Science Tools, Heidelberg, Germany) following by midsagittal division of hemispheres. 400 µm transversal slices were cut on a Mcllwain tissue chopper (Quantum Design GmbH, Darmstadt, Germany). Complete and undamaged hippocampal sections were selected for subsequent culturing in 6-well plates. 5 slices were placed in each well onto 0.4 µm membrane inserts (PICM03050, Merck Millipore, Darmstadt, Germany) with 1 ml of culturing medium (MEM, 32360034, Thermo Fisher Scientific), supplemented with 20% horse serum (26050088, Thermo Fisher Scientific), 1 mM GlutaMAX (35050061, Thermo Fisher Scientific), 0.000125% ascorbic acid (A92902, Sigma), 0.001 mg/ml insulin (I2643, Sigma), 1 mM CaCl_2_, 2 mM MgSO_4_, 20 mM D-glucose (all Carl Roth GmbH + Co. KG), and 100 U/ml penicillin/streptomycin (15140122, Thermo Fisher Scientific). Medium was changed after 3 h and subsequently every 3 days for up to 14 days in vitro (div). Cultures were maintained in a humidified HeraCell150 Incubator (Thermo Fisher Scientific) at 37 °C with 5% CO_2_. Slices underwent flattening during the first 10 days and appeared transparent with clearly visible hippocampal compartments until 14 div.

### Dissociated hippocampal neuronal cultures

Primary hippocampal neurons (PHN) were prepared and cultured from P0 Slack^+/+^ and Slack^−/−^ pups of both sexes according to the established protocol described below^22^. Pups were decapitated and brains transferred to ice-cold dissecting medium (HBSS, 10 mM HEPES, 1 mM sodium pyruvate (Thermo Fisher Scientific, 0.1% D-glucose)). Hippocampi were isolated as described for HSC and freed from meninges. After washing in dissecting medium, hippocampi were incubated with trypsin (0.25%, 15090, Thermo Fisher Scientific) at 37 °C for 20 min before 0.1% Desoxyribonuclease I (DN25, Sigma Aldrich Chemie GmbH) was added for another 5 min at room temperature. Next, hippocampi were washed with dissecting and plating medium (BME with EBSS, 41010-026, Thermo Fisher Scientific), 10% fetal bovine serum (16140071, Thermo Fisher Scientific; 0.45% D-glucose, 1 mM sodium pyruvate and 2 mM GlutaMAX, 100 U/ml P/S). Hippocampi were dissociated by gentle trituration in plating medium with fire-polished Pasteur pipets and subsequently seeded onto poly-L-Lysine coated coverslips (0.5 mg/ml, P2636, Sigma Aldrich Chemie GmbH). 110,000 cells in plating medium were either plated on 32 mm diameter coverslips restricted by silicon culture inserts (80466, ibidi GmbH, Graefelfing, Germany) to reduce growth area, or on 12 mm coverslips. 2 h after seeding, medium was changed to maintenance medium (Neurobasal, 21103049, Thermo Fisher Scientific with B-27, 17504044, Thermo Fisher Scientific and 2 mM GlutaMAX) and kept in culture for 8 to 14 days by changing 30% of medium every 4 days.

### Propidium iodide-based cell death assay

KA-induced cell death in HSC and PHN was assessed using propidium iodide (PI) staining. Basal viability of 14 div HSC was measured after 10 min incubation with 2 µg/ml PI (P4864, Sigma Aldrich Chemie GmbH) using an RFP filter block in a Nikon Eclipse Ts2R microscope (Nikon Instruments Inc., Melville, US) Plan Fluor OFN25 ×4 objective (Nikon Instruments Inc.) and a DMK 33Ux174 camera (oem cameras, Middletown, US). PI uptake was measured 24 h after the addition of 5 or 10 µM KA to the medium. To provide a KA-independent positive control at the end of each experiment, culturing medium was replaced by 1 ml of 80% EtOH for 1 h at −20 °C before new PI was re-applied for a final set of images. Relative PI uptake was quantified as described^34^ by using Fiji^35^). Three circular regions of interest (ROI, 80 × 80 pixels) were placed on each CA1/2, CA3/4, and dentate gyrus. An additional tenth region was placed adjacent to the slice for background subtraction. Background subtracted integrated densities of each region were averaged for each hippocampal compartment. KA-induced PI uptake was calculated relative to the positive control for each slice following the subtraction of basal PI uptake.

For cell death detection of dissociated neurons, PHN were plated on ibidi 8 well chamber slides (80826, ibidi GmbH) and cultured for 8 to 14 div. For measurement, PI (2 µg/ml) and KA (5 or 10 µM) were added to each well. In a second set of experiments, PHN was treated for 24 h with 10 µM KA and 50 or 100 µM picrotoxin (PiTX). Slides were placed into a prewarmed stage top incubation system (10722, 11922-DL, 10918-DL, ibidi GmbH) maintaining 5% CO_2_, 21% O_2_, and 80% humidity at 37 °C. For each well, time courses from 4 to 5 ROI were automatically imaged using a BioPrecision2 automated XY-table (Ludl Electronic Products Ltd., Hawthorne, US) on a Zeiss Axio Observer Z1 inverted microscope equipped with a Zeiss EC-Plan-Neofluar ×20/0.5 objective (440340-9904, Carl Zeiss AG, Oberkochen, Germany) and a LedHUB LED light-engine equipped with 505–600 nm LED (Omicron Laserage Laserprodukte GmbH, Dudenhofen, Germany). Filter set (475/543/702 nm) was obtained from AHF Analysetechnik. PI emission was automatically detected every 30 min for 24 h with a PCO panda 4.2bi camera (Excelitas PCO GmbH, Kelheim, Germany) controlled by VisiView software (Visitron Systems GmbH, Puchheim, Germany). Fiji was used to count particles in each ROI over time after background subtraction and application of a constant threshold to calculate relative PI uptake to basal number of particles.

### Immunofluorescence staining

Slack expression and maturity of dissociated hippocampal cultures were verified by staining against MAP2 at 8 and 14 div. First, cells were washed twice with warm HBSS and fixed for 10 min with warm fixation solution (DPBS, 14190-094, Thermo Fisher Scientific with, 4% Paraformaldehyde and 4% sucrose (Carl Roth GmbH + Co. KG)). Cells were washed twice and incubated for 2 h at room temperature with blocking solution (DPBS with 2% Glycerol, 0.3% Triton X-100, 50 mM NH_4_Cl, 5% NGS Vector Labs S-1000, 2% BSA (0163.2, Carl Roth GmbH + Co. KG)). Subsequently, cells were incubated with primary antibodies (1:300 monoclonal anti-KCNT1, SAB5200036, Sigma Aldrich Chemie GmbH, 1:1500 monoclonal MAP2, D5G1, Cell Signaling Technology, Leiden, Netherlands) in blocking buffer for 24 h at 4 °C. Next, cells were washed in washing solution (DPBS with 0.01% Triton X-100) and incubated for 2 h at room temperature with secondary antibodies (Alexa555, A21127, Alexa488, A11034, Thermo Fisher Scientific) and Hoechst 33342 (1:1000) in blocking buffer. Cells were washed in washing solution, DPBS, and water. Cells were mounted with PermaFluor aqueous mounting medium (TA-030-FM, Thermo Fisher Scientific) and imaged the next day with a Zeiss Axiovert 200 M equipped with a color camera (AxioCam MRc Rev 3) and ZEN 3.4 software (Carl Zeiss AG).

### Ca^2+^ imaging

8 to 14 div PHN loaded with 2.5 µM Fura-2AM (21021, Biomol GmbH, Hamburg, Germany) in maintenance medium for 40 min and were subsequently transferred to a PC30 perfusion chamber (NGFI GmbH, Graz, Austria) connected to a gravity-based perfusion system (NGFI GmbH) to obtain constant perfusion with prewarmed recording buffer (in mM: 138 NaCl, 5 KCl, 2 CaCl_2_, 1 MgCl_2_, 10 HEPES, 10 D-glucose). Intracellular Ca^2+^ concentration ([Ca^2+^]_i_) was measured using a Zeiss Axiovert 200 equipped with a Zeiss Fluar 440255 ×40/1.30 oil immersion objective (Carl Zeiss AG) and illuminated by a CoolLED *p*E-340^fura^ (CoolLED Ltd, Andover, US). The light was filtered by AHF Analysetechnik F39-380 and F39-343 nm BrightLine, passed the dichroic filter AT515LP, and the emission was finally filtered by CmF 525/15. Fura-2 was imaged at 1 Hz, with a binning of 4 using a PCO panda 4.2 camera (Excelitas PCO GmbH) and VisiView software (Visitron Systems GmbH) with background correction. After 2 min measurement of basal [Ca^2+^]_i_, PHN was superfused for 2 min with recording buffer containing 1 to 100 µM KA before washout for 3 min. Maximum change in fluorescence emission ratio between excitation at 340 nm and 340 nm was calculated. All cells from one recording were averaged and data was analyzed using GraphPad Prism 8 (GraphPad Software, Boston, US).

### K^+^ imaging

After 8–14 div PHN were virally transduced with an adeno-associated virus-DJ/8 vector system encoding the cytosol targeted K^+^ sensitive, FRET-based biosensor NES lc-LysM GEPII 1.0^36^ under control of a CAG promoter at a multiplicity of infection (MOI) of 100. PHN were imaged 72 h after transduction in a PC30 perfusion chamber (NGFI GmbH) under constant perfusion with prewarmed imaging buffer (in mM: 126.5 NaCl, 5 KCl, 2 CaCl_2_, 2 MgCl_2_, 10 HEPES, 30 D-glucose, 10 sodium pyruvate) by a gravity-based perfusion system (NGFI GmbH). Imaging was performed using a Zeiss Axio Observer Z1 inverted microscope equipped with a Zeiss EC Plan-NEOFLUAR ×40/1.3 Oil 420460-9900 objective (Carl Zeiss AG) and connected to a LedHUB LED light-engine producing excitation light at a wavelength of 430 nm (Omicron Laserage Laserprodukte GmbH). The filter set was obtained from AHF Analysetechnik for 427/10 nm. Emission light of GEPII 1.0 was collected simultaneously at 475 and 530 nm using an Optosplit II optical image splitter (Cairn Research, Faversham, UK) equipped with a T505Ipxr (AHF Analysentechnick) for CFP/YFP connected to a PCO panda 4.2bi camera (Excelitas PCO GmbH). Images were acquired at 1 Hz with a binning of 4 using Visiview software (Visitron Systems GmbH). Ratios were calculated after background correction by YFP to CFP division and the ratio was normalized for first 2 min of baseline recording to obtain maximal changes in YFP/CFP ratios during KA stimulations. All cells from one recording were averaged and further data analysis was performed using GraphPad Prism 8 (GraphPad Software).

### Electrophysiology

Coverslips with 14 div PHN were transferred to a submerged-type recording chamber (Warner Instruments USA) constantly perfused with extracellular buffer (in mM: 140 NaCl, 2.5 KCl, 2 CaCl_2_, 4 MgCl_2_, 10 HEPES, 10 D-glucose, pH 7.4, 300 mOsm/kg). Cells were visualized by a Nikon Tc2R equipped with a Nikon S Plan Fluor ×40/0.6 objective with EMBOSS contrast and a DFK 33Ux174 camera (Nikon Instruments Inc.). 3.5–4 MΩ micropipettes were pulled from borosilicate glass (BM150-10P, Science Products GmbH, Hofheim, Germany) using a P-1000 Micropipette Puller (Sutter Instruments, Novato, US) and polished by a MF-830 Micro Forge (Narishige International Ltd., London, UK) and filled with intracellular buffer (in mM: 136 K-gluconate, 0.6 MgCl_2_, 17.8 HEPES, 1 EGTA, 4 Mg-ATP, 0.3 Na^2^-GTP, pH 7.4, 300 mOsm/kg). Whole-cell recordings were sampled at 5 kHz using an EPC10 amplifier (HEKA Elektronik GmbH, Lambrecht, Germany) and PatchMaster software. Whole-cell capacitance and series resistance were compensated. Cells with changes in access resistance exceeding 20% during recording were excluded from the analysis. Data was analyzed using FitMaster software. Cells were held at −60 mV and whole-cell current responses to 500 ms voltage steps between −60 to +80 mV in 20 mV increments were recorded before and after perfusion with 10 µM tetrodotoxin (TTX, Carl Roth GmbH + Co. KG). Minimum current amplitude in a 15 ms window at the beginning and mean amplitude in the last 25 ms of the depolarizing pulse were used to measure transient inward currents and steady-state currents, respectively.

5 µM KA were added to neurons held in current-clamp mode near −60 mV. Starting from the first KA-induced AP, the number of APs per 1 s bin was counted for 30 s as well as AP threshold (membrane potential at AP initiation) and amplitude (from threshold to peak). For current injections, cells were held near −60 mV in current-clamp mode and depolarized by 10 current injections in increments of 20 pA for 500 ms. AP number per depolarization as well as AP threshold (membrane potential at AP initiation), AP amplitude (from threshold to peak) and afterhyperpolarization (aHP) duration (time from HP peak to resting potential^37^), and amplitude (aHP peak minus resting potential) were measured for the first AP. Maximal AP rise slope, maximal AP decay slope, and AP halfwidth were analyzed using threshold-based event-detection of Clampfit 10.7 (Molecular Devices LLC).

### Quantitative RT-PCR

Hippocampi were isolated 24 h after KA-induced seizures, homogenized (Polytron PT 1200E), and extracted in 1 ml Peq-GOLD RNApure (VWR International GmbH, Darmstadt, Germany) according to manufacturer’s instructions. mRNA was extracted from 20 pooled hippocampal brain slice cultures using the NucleoSpin kit (MACHEREY-NAGEL GmbH & Co. KG, Düren, Germany) according to manufacturer’s instructions. Genomic DNA was digested using DNase I (04716728001, Sigma Aldrich Chemie GmbH,) at 54 °C for 30 min. 500 ng RNA was reversely transcribed by iScript cDNA Synthesis Kit (170-8896, Bio-Rad Laboratories GmbH, Feldkirchen, Germany). Real-time quantitative PCR (RT-PCR) was performed in triplicates with 7.5 ng/µl mRNA and 333 nmol/l of corresponding primers. Each run included water and reverse transcriptase (RT) negative controls (where RT was omitted during reverse transcription). Reactions were performed using SSoAdvanced Universal SYBR Green Supermix (1725274, Bio-Rad Laboratories GmbH) on a CFX Connect Real-Time PCR Detection System (Bio-Rad Laboratories GmbH) by incubating at 92 °C for 2 min, followed by 40 cycles of 5 s at 95 °C and 30 s at 58 °C. Expression of targeted genes was calculated by the 2^−∆∆Ct^ method relative to hypoxanthine-guanine phosphoribosyl transferase (HPRT). Primer sequences were designed by Primer3 and PrimerBLAST software and are listed in Supplementary Table 1.

### Statistics and reproducibility

**Fig. 1:**
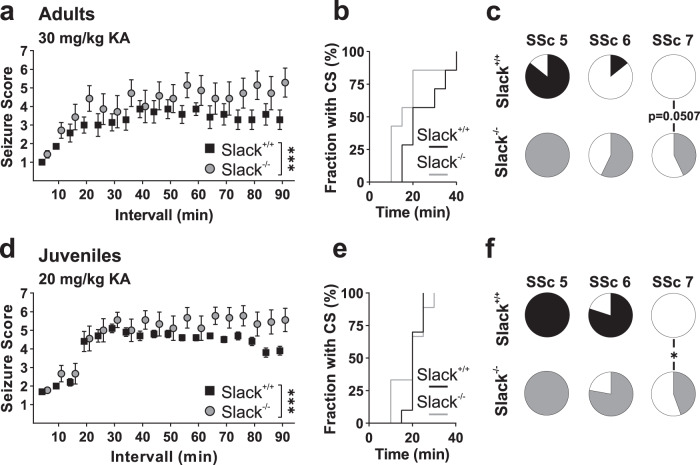
Increased severity and lethality of kainic acid-induced seizures in Slack^−/−^. **a** 12 weeks-old adult male wildtype (Slack^+/+^) and knockout (Slack^−/−^) mice were tested for seizure susceptibility and severity by injection of 30 mg/kg KA i.p. Seizures were scored using an adopted Racine Scale (from Seizure Score 0 (SSc 0), no seizures, to SSc 6, SE, and SSc 7, death after SE). Compared to Slack^+/+^ (*N* = 7), Slack^−/−^ (*N* = 7) animals display significantly (*p* < 0.001; two-way ANOVA F_1,216_ = 24.32) increased seizure severity over time, with **b** a similar onset of convulsive seizures (CS, SSc ≥3) but **c** more frequent occurrence of tonic-clonic seizure (SSc 6) and almost significantly more frequent death (SSc 7) (*χ*², *p* = 0.0507) following SE (black and gray sectors in **c** represent the affected, white sectors the unaffected fraction of animals by genotype for each respective SSc). Fractions of animals with SSc ≤ 5 were similar for both genotypes. **d** 4-week-old juvenile mice were tested for seizure susceptibility and severity by i.p. injection of 20 mg/kg KA and scoring. Compared to Slack^+/+^ (*N* = 10), Slack^−/−^ (*N* = 9) animals display significantly (*p* < 0.001; two-way ANOVA F_1,144_ = 30.63) increased seizure severity over time with **e** similar onset of CS but **f** significantly more frequent death (SSc 7) (*χ*², *p* = 0.017) following SE (black and gray sectors in **f** represent the affected, white sectors the unaffected fraction of animals by genotype for each respective SSc). Response to SSc ≤ 5 was similar for both genotypes. Data represented as mean ± SEM. For detailed statistics also consult Supplementary Data 1.

Data were analyzed using GraphPad Prism version 8 (GraphPad Software). First, D’Agostino & Pearson test for normality distribution was performed, followed by the appropriate outlier test. Genotype-dependent effects were analyzed by unpaired t-test including Welch’s correction in case of different variance, Mann-Whitney test in case of non-parametric data, and two-way ANOVA in case of time course-related data. Juvenile mice also died following KA-induced seizures, so mRNA transcript levels were analyzed between surviving and dead animals. Since no genotype-specific effects were found, all Slack^−/−^ samples were pooled. Fraction of animals that reach different SSc upon KA injection was reported as part of the whole and in percent (Fig. 1c, f). Differences were statistically assessed by *χ*² test, although its meaningfulness is somewhat limited for small numbers^38^. In the case of Fura-2 measurements with increasing KA concentrations, a non-linear, sigmoidal curve fit was performed to determine the logEC_50_ and goodness of curve fit. Sample size (*N* value) is defined as number of animals for KA-based epilepsy testing. For quantification of KA-induced PI uptake in HSC n was defined as mean of three regions placed in each, CA1, CA3, and DG of 5 slices cultured together in one well. Slices of 3 to 6 preparations were analyzed. For quantification of KA-induced PI uptake in PHN, sample size n was defined as ROI with four to five ROI per well and recording. Recordings were performed from three preparations. For Fura-2-based Ca^2+^ measurements and FRET-based K^+^ measurements cells of each measurement were averaged to one experimental sample *n*. Data are acquired from 8 to 10 preparation and 12 to 13 wells. For patch-clamp experiments, each cell was defined as one sample *n* and data analysis based on 6 to 7 preparations for voltage-clamp data and 8 to 9 preparations for current-clamp data. All data are represented as mean ± SEM. In figures, significance is indicated by asterisks (*p < 0.05, **p < 0.01, ***p < 0.001). n.s. denotes non-significant results (*p* > 0.05). Statistics are listed together with raw data in Supplementary Data 1.

### Reporting summary

Further information on research design is available in the Nature Portfolio Reporting Summary linked to this article.

## Results

### Slack^−/−^ mice are more susceptible to kainic acid-induced acute epilepsy

First, we aimed to elucidate Slack’s role in acute seizures by applying the widely studied KA-based model. Seizures were provoked by injection of 30 mg/kg KA in 12 weeks-old male mice. Animals were observed over 90 min and scored in 5 min intervals using an adopted Racine Scale. Within 20 min, KA injection provoked convulsive behavior with seizure score (SSc) 3 and higher in both genotypes. Overall SSc was significantly increased in Slack^−/−^ compared to Slack^+/+^ (Fig. 1a). Time to reach convulsive seizures (CS) with a SSc of ≥3 was similar for both genotypes (Fig. 1b). Additionally, the fractions of Slack^−/−^ developing SE (Slack^+/+^: 1 of 7; Slack^−/−^: 4 of 7) was increased but this difference was not significant (*p* = 0.09) while there was a clear tendency (*p* = 0.05) for a higher probability of Slack^−/−^ (Slack^+/+^: 0 of 7; Slack^−/−^: 3 of 7) to die from seizures (Fig. 1c). Since Slack-related epilepsy syndromes in human carriers of pathogenic *KCNT1* variants are characterized by early onset, we also injected 4 weeks-old juvenile male mice with 20 mg/kg KA. Juvenile mice of both genotypes responded with severe convulsions and reached SSc ≥4 within 20 min after KA application (Fig. 1d), with a similar time of onset of CS (Fig. 1e). While the fraction of animals reaching SE (Slack^+/+^: 8 of 10; Slack^−/−^: 7 of 9) was not different, Slack^−/−^ showed significantly increased seizure-induced lethality (Slack^+/+^: 0 of 10; Slack^−/−^: 4 of 9) compared to Slack^+/+^ (Fig. 1f). Interestingly, by analyzing mRNA expression levels of related channel subunits 24 h following KA-induced seizures, no prominent alterations were found in either genotype (Supplementary Fig. 1a–f), despite a significantly decreased level of BDNF in Slack^−/−^ (Supplementary Fig. 1f). These results demonstrate that KA-induced seizures are more severe in adult and juvenile Slack^−/−^, which suggests that Slack channels acutely limit epileptic neuronal activity in vivo.

### Kainic acid amplifies cell death in Slack^−/−^

**Fig. 2:**
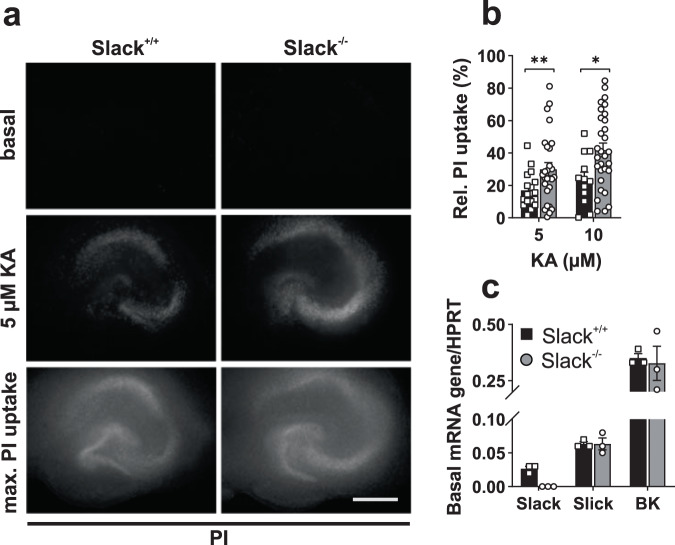
Increased KA-induced cell death in Slack^−/−^ organotypic hippocampal slice cultures. **a** Representative images of 14 div slice cultures from Slack^+/+^ and Slack^−/−^ before (basal) treatment, after 24 h treatment with 5 µM KA, and after application of 80% ethanol (max. PI uptake). Scale bar: 500 µm. **b** Compared to Slack^+/+^, Slack^−/−^ slice cultures show significantly increased PI uptake (normalized to maximum) in response to 24 h treatment with 5 µM (Slack^+/+^ *n* = 18 ROI out of four preparations, Slack^−/−^ *n* = 27 ROI out of six preparations, unpaired *t* test with Welch’s correction *p* = 0.009) and 10 µM KA (Slack^+/+^ *n* = 12, Slack^−/−^ *n* = 24, unpaired *t* test *p* = 0.016). **c** mRNA from 14 div Slack^+/+^ and Slack^−/−^ hippocampal slice cultures, isolated for quantitative RT-PCR analysis. No Slack mRNA is detected in Slack^−/−^. Slick and BK mRNA expression levels are similar in Slack^+/+^ and Slack^−/−^. Data in **b**, **c** represented as mean ± SEM with *p* < 0.05 and *p* < 0.01. For detailed statistics also consult Supplementary Data 1.

The limbic system, especially the hippocampal formation is prone to be focused for ictal events and epileptic seizures, leading to hippocampal sclerosis and temporal lobe epilepsies^39^. Furthermore, Slack channels^21^ and KA receptors (GluK)^40,41^ are also highly expressed in the hippocampus and KA injection is known to produce neuronal cell death in different hippocampal layers. We therefore assessed sensitivity to KA-induced cell death of hippocampal neurons in vitro by quantifying propidium iodide (PI) uptake. First, we used HSC, a model in which physiological intrahippocampal synaptic connections remain preserved^42^. 24 h exposure of isolated HSCs to 5 or 10 µM KA significantly increased cell death in cultures from Slack^−/−^ compared to Slack^+/+^ (Fig. 2a, b). PI uptake seemed to be highest in CA3 (see Fig. 2a middle, Supplementary Fig. 2), which is the hippocampal area with the highest GluK expression as well as the one that is most susceptible to degeneration due to hippocampal sclerosis and temporal lobe epilepsy^39^. Next, we tested if the effects observed in cultures from Slack^−/−^ might be due to expressional changes of closely related K^+^ channels. Transcript levels of the related and highly homologous Na^+^-, or Ca^2+^-activated K^+^ channels Slick and BK, however, were unchanged between both genotypes in mature HSC, contradicting compensatory regulation of these two K^+^ channels under pathophysiological conditions (Fig. 2c).

**Fig. 3:**
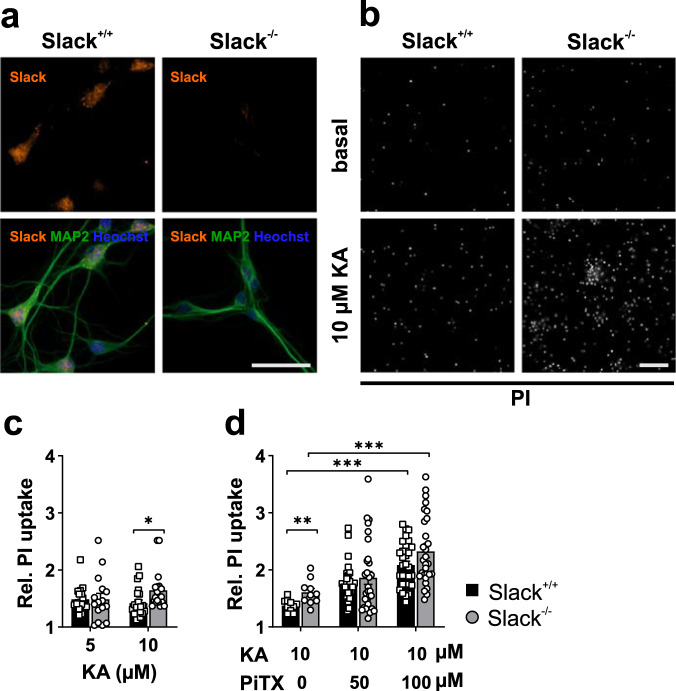
Increased KA-induced cell death in Slack^−/−^ dissociated hippocampal neurons. **a** Representative fluorescence images of 8 div hippocampal Slack^+/+^ and Slack^−/−^ neurons stained with specific antibodies against Slack (red) and MAP2 (green). Nuclei were visualized with Hoechst 33342 (blue). Slack immunoreactivity is not detected in Slack^−/−^. Scale bar: 40 µm. **b** Representative images of PI uptake in 8 div hippocampal neurons before (basal, top) and after 24 h exposure to 10 µM KA (bottom). Scale bar: 500 µm. **c** Cell death is significantly increased (Mann–Whitney test, *p* = 0.0016) in Slack^−/−^ (*n* = 22 ROI out of three preparations) compared to Slack^+/+^ (*n* = 20 ROI out of three preparations) as measured by PI uptake (normalized to basal) after 24 h exposure to 10 µM KA. **d** PI uptake in response to 10 µM KA is significantly amplified by 24 h co-treatment with 100 µM PiTX (Slack^+/+^ *n* = 30 ROI out of three preparations, Slack^−/−^ *n* = 30 ROI out of three preparations, Sidak’s multiple comparison *p* = 0.0008 for Slack^+/+^, *p* = 0.0007 for Slack^−/−^). Slack^−/−^ PHN are overall more affected than Slack^+/+^ (two-way-ANOVA, F_1,134_ = 4.270, *p* = 0.040). Data in **c** represented as mean ± SEM with *p* < 0.05, *p* < 0.01 and *p* < 0.001. For detailed statistics also consult Supplementary Data 1.

Subsequently, we analyzed KA-induced cell death in 8 to 10 div cultures from dissociated Slack^+/+^ and Slack^−/−^ hippocampal neurons to observe cell-autonomous effects independent from intrahippocampal synaptic connections. MAP2-expressing 8–10 div hippocampal Slack^+/+^ neurons show robust Slack immunoreactivity which is absent in Slack^−/−^ (Fig. 3a). Subsequently, neuronal cultures were again exposed to either 5 or 10 µM KA over 24 h. In accordance with our findings in HSC, cell death in Slack^−/−^ neurons exposed to 10 µM KA was significantly increased in comparison to Slack^+/+^ (Fig. 3b, c). To assess whether this observed cell death is due to increased neural activity, we co-treated PHN with 50 or 100 µM of the GABA_A_ inhibitor picrotoxin (PiTX) to increase neuronal excitability. As expected, PiTX dose-dependently led to significantly amplified KA-induced cell death in both genotypes, indicating excessive neuronal activity to drive neuronal demise (Fig. 3d). Under conditions of simultaneous treatment with KA and PiTX, however, cell death was not significantly different between genotypes within the individual conditions, but on a group level (see Supplementary Data 1). Taken together, increased cell death in Slack^−/−^ HSC and dissociated hippocampal neuron cultures provide in vitro confirmation of Slack’s neuroprotective role, which was initially revealed by in vivo experiments.

### Kainic acid-induced Ca^2+^ influx and K^+^ efflux is increased in Slack^−/−^ hippocampal neurons

**Fig. 4:**
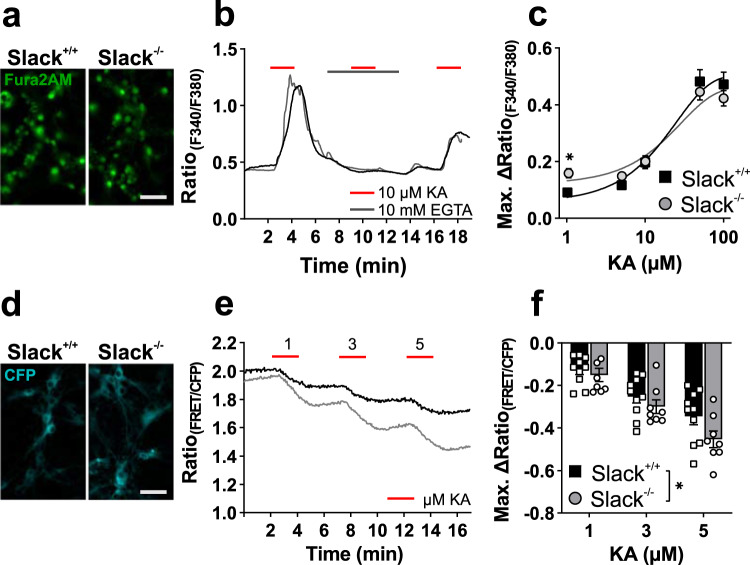
Increased KA-induced Ca^2+^ influx and K^+^ efflux in Slack^−/−^ hippocampal neurons. **a** Representative images of Fura-2-loaded 8 div hippocampal neurons illuminated at 380 nm. Scale bar: 50 µm. **b** Representative time course of Fura-2 recordings in hippocampal neurons treated with 10 µM KA (red bar) alone or in presence of 10 mM EGTA (black bar). **c** Maximum change in ratio between fluorescence emission upon excitation at 340 nm and 380 nm in response to increasing KA concentrations. Concentration-response curves did not reveal differences between genotypes (Slack^+/+^ *n* = 12–32, EC_50_ = 13.56, 95% IC = 9.14 to 20.11, *R*^2^ = 0.75; Slack^−/−^ *n* = 13–27, EC_50_ = 13.78, 95% IC = 8.92 to 21.29, *R*^2^ = 0.72). Scale bar: 50 µm. At a low concentration of 1 µM KA, Slack^−/−^ (*n* = 32) responded with significantly increased Ca^2+^ influx compared to Slack^+/+^ (Sidak’s multiple comparison *p* = 0.013, *n* = 27). **d** Representative 8 div hippocampal neurons virally transduced with the FRET-based K^+^-sensitive sensor (GEPII). Scale bar: 50 µm. **e** Representative time course of [K^+^]_i_ recording by NES lc-LysM GEPII 1.0 based FRET/CFP ratio in hippocampal neurons treated with indicated KA concentrations. **f** [K^+^]_i_ was significantly (two-way ANOVA, F_1,48_ = 4.8, *p* = 0.031) reduced in Slack^−/−^ (*n* = eight wells out of five preparations) compared to Slack^+/+^ (*n* = 10 wells out of six preparations) neurons following treatment with 1, 3, and 5 µM KA. Data of **c**, **f** represented as mean ± SEM with *p* < 0.05. For detailed statistics also consult Supplementary Data 1.

In order to elucidate the molecular processes underlying the increased vulnerability of Slack^−/−^ animals to KA-induced seizures and increased demise of Slack^−/−^ cells after KA stimulation, we performed single-neuron live recordings of [Ca^2+^]_i_ and [K^+^]_i_ concentrations in the presence or absence of KA. KA-induced [Ca^2+^]_i_ increases were observed after perfusion of 8 to 10 div hippocampal neurons loaded with Fura-2AM (Fig. 4a). Robust abrogation of the Ca^2+^ signal by extracellular EGTA verified the extracellular space as source for the KA-stimulated [Ca^2+^]_i_ increase (Fig. 4b). Subsequently, PHN were perfused with KA concentrations from 1 to 100 µM, and maximal delta ratios observed in Fura-2 emission ratios fitted a sigmoidal concentration-response curve (Fig. 4c). EC_50_ values did not differ between Slack^+/+^ (EC_50_ = 13.56 µM) and Slack^−/−^ (EC_50_ = 13.78 µM) indicating similar GluK densities in neurons from both genotypes, while [Ca^2+^]_i_ of Slack^−/−^ appeared to be increased at 1 µM KA (*p* = 0.002).

To investigate the role of Slack-mediated K^+^ currents during KA stimulation, we monitored [K^+^]_i_ in 8–10 div hippocampal neurons transduced with the genetically encoded potassium sensor NES lc-LysM GEPII 1.0 (Fig. 4d). Interestingly, stimulation with 1, 3 and 5 µM KA provoked significantly stronger reduction of [K^+^]_i_ in Slack^−/−^ than in Slack^+/+^ (Fig. 4e, f). Taken together, live-cell imaging data suggest that KA stimulation leads to increased Ca^2+^ influx as well as increased K^+^ efflux in Slack^−/−^ neurons. Even though the latter result was somewhat counterintuitive.

### Slack^+/+^ hippocampal neurons display Slack-specific TTX-sensitive outward currents and limited excitability

**Fig. 5:**
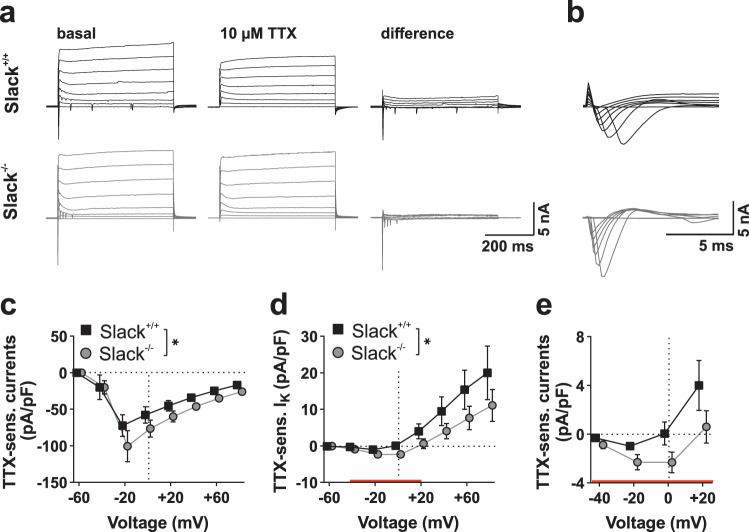
Increased Na^+^ currents in Slack^−/−^ hippocampal neurons. **a**–**e** Voltage-clamp recordings of whole-cell currents from 14 div hippocampal neurons. **a** Representative recordings from Slack^+/+^ and Slack^−/−^ neurons responding to depolarizing voltage steps (from −60 to +80 mV in 20 mV increments) before (left), after perfusion with TTX (middle) and following digital subtraction (right). **b** (Magnification from **a**) Depolarization-induced, TTX-sensitive transient inward currents. **c** Compared to Slack^+/+^ (*n* = 20 from six preparations), TTX-sensitive inward current amplitudes in Slack^−/−^ (*n* = 17 from 7 preparations) neurons were significantly (*p* < 0.0168) increased (two-way ANOVA, F_1,280_ = 5.791). **d** TTX-sensitive steady-state outward currents were significantly (two-way ANOVA, F_1,312_ = 5.122, *p* = 0.0243) higher in Slack^+/+^ (*n* = 24) compared to Slack^−/−^ (*n* = 17). **e** (Magnification of red section from **d**) TTX-sensitive steady-state currents at voltage steps from −40 to +20 mV indicated an enlarged inward-directed current component in Slack^−/−^ probably representing *I*_NaP_. Data represented as mean ± SEM with *p* < 0.05. For detailed statistics also consult Supplementary Data 1.

We next examined whether the changes in Ca^2+^ and K^+^ homeostasis observed in Slack^−/−^ neurons translate into altered ion conductances and neuronal excitability. To this end, we first performed voltage-clamp recordings of 14 div PHN to observe whole-cell currents in response to depolarizing voltage steps. Tetrodotoxin (TTX), a blocker of voltage-gated Na^+^ channels, was applied to isolate currents conducted by Na^+^ channels as well as Na^+^-dependent K^+^ channels (Fig. 5a, b). Depolarization-induced transient inward current densities were significantly higher in Slack^−/−^ than Slack^+/+^ neurons (Fig. 5b, c). Additionally, we observed significantly altered TTX-sensitive steady-state current densities between both genotypes (Fig. 5d). This difference is due to reduced outward current densities at depolarized membrane potentials, likely representing the lack of Na^+^-activated K^+^ currents in Slack^−/−^. Additionally, we also noticed a pronounced inward deflection of Slack^−/−^ steady-state currents between −40 and +20 mV (Fig. 5d with red line and e). This might be due to the increased expression of an inward-directed current component in Slack^−/−^, likely persistent Na^+^ currents (I_NaP_), which were previously observed in *Drosophila* neurons lacking the Slack analog Slo2^43^ as well as in mammalian neurons^11,17^. These findings indicate that Slack^−/−^ neurons lack TTX-sensitive K^+^ currents and also display changes in inwardly directed current components.

**Fig. 6:**
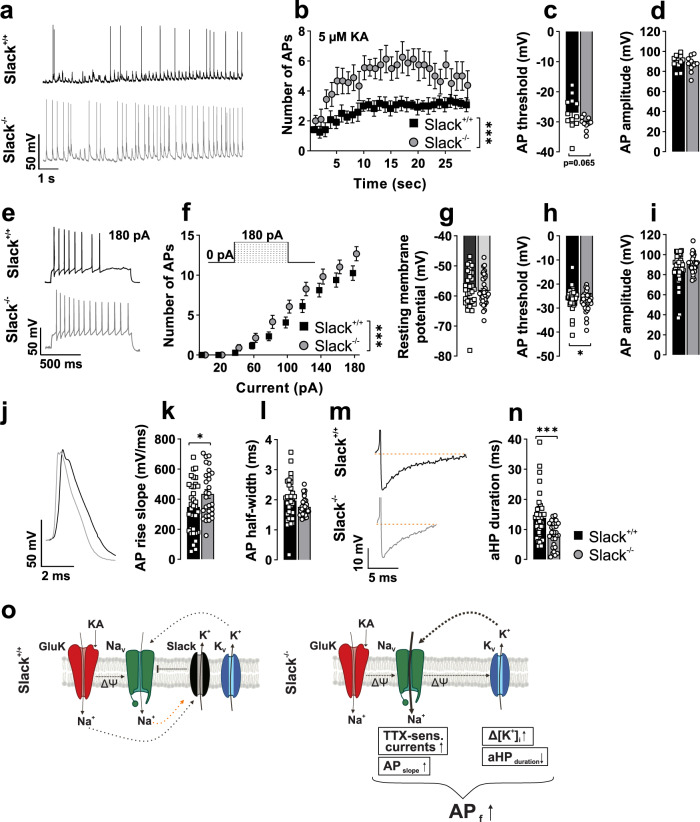
Increased action potential frequencies in Slack^−/−^ hippocampal neurons. **a**–**n** Whole-cell current-clamp recordings from 14 div hippocampal neurons. **a** Representative current-clamp recordings from Slack^+/+^ and Slack^−/−^ neurons after application of 5 µM KA. **b** Number of AP per 1 s bin after KA application was significantly (two-way ANOVA, F_1,551_ = 175.4, *p* < 0.001) increased in Slack^−/−^ (*n* = 12 independent experiments from a total of *n* = 12 neurons from 6 preparations) compared to Slack^+/+^ (*n* = 9 from 4 preparations), while **c** action potential (AP) threshold (Welch’s corrected unpaired t-test) and **d** amplitude were not significantly different. **e** Representative current-clamp recordings from Slack^+/+^ (top) and Slack^−/−^ (bottom) neurons during 180 pA current injection. **f** AP frequency in response to incremental current injection was significantly (two-way ANOVA F_1,668_ = 20.50, *p* < 0.001) increased in Slack^−/−^ (*n* = 33 neurons obtained from 8 preparations) compared to Slack^+/+^ cells (*n* = 38 neurons obtained from nine preparations). **g** Resting membrane potential was not different between genotypes. **h**, **i** Slack^−/−^ neurons displayed significantly (Mann–Whitney test *p* = 0.043) more negative threshold potential for initiation of AP firing than Slack^+/+^ neurons together with similar AP amplitude. **j** Representative current-clamp recording illustrating different AP kinetics between Slack^+/+^ (black) and Slack^−/−^ (gray). **k** Maximal AP rise slope is significantly (Mann–Whitney test *p* = 0.041) steeper in Slack^−/−^ (*n* = 29) compared to Slack^+/+^ (*n* = 34), while **l** AP halfwidth is similar between genotypes. **m** Representative current-clamp recordings illustrating different AP afterhyperpolarization (aHP) between Slack^+/+^ (black) and Slack^−/−^ (gray). **n** aHP duration is significantly (Mann–Whitney test *p* = 0.0009) shorter in Slack^−/−^ than Slack^+/+^ neurons. **o** Schematic illustration of proposed Slack function during KA-induced neuronal activation in Slack^+/+^ (left) and Slack^−/−^ (right). Lack of subthreshold Slack activity leads to disinhibition of Na_V_ channels to reduce AP threshold, which, in turn, increases AP frequency and presumably boosts K_V_ channel activation. Data represented as mean ± SEM with *p* < 0.05 and *p* < 001. For detailed statistics also consult Supplementary Data 1.

To examine, if the observed changes in TTX-sensitive currents influence neuronal activity, we performed current-clamp recordings of 14 div PHN during bath application of KA. AP frequencies in 1 s bins recorded from the first AP after application of 5 µM KA were significantly increased in Slack^−/−^ compared to Slack^+/+^ (Fig. 6a, b). This effect was accompanied by AP generation at tendentially more negative membrane potentials in Slack^−/−^ (Fig. 6c), while AP amplitude remained unchanged between genotypes (Fig. 6d). Injection of depolarizing currents also provoked significantly higher AP frequencies for a given current amplitude in Slack^−/−^ than Slack^+/+^ (Fig. 6e, f). This increased excitability cannot be explained by an altered resting membrane potential (Fig. 6g). It might, however, be due to initiation of AP at significantly more negative membrane potentials in Slack-deficient neurons (Fig. 6h). Although peak amplitudes of the elicited AP or aHP were not different between genotypes (Fig. 6i and Supplementary Data 1), AP rise slope was significantly steeper in Slack^−/−^ neurons (Fig. 6j, k) while AP halfwidth was similar between genotypes (Fig. 6l). These changes in AP kinetics are congruent with the observed amplification of TTX-sensitive inward currents in Slack^−/−^ (Fig. 5). Additionally, Slack^−/−^ neurons showed a significantly shorter aHP duration resulting in earlier return to resting potential compared to Slack^+/+^, which implies that Slack K^+^ channels limit neuronal firing patterns by prolonging aHP duration (Fig. 6m, n).

In summary, Slack deficiency alters densities of inwardly and outwardly directed TTX-sensitive conductances, the threshold potential for AP initiation, and shortens aHP duration to accelerate KA- and depolarization-induced AP firing frequencies.

## Discussion

Here we demonstrate that adult and juvenile Slack^−/−^ mice suffer from increased severity of KA-induced seizures (Fig. 1). This finding is consistent with previously documented epileptogenic LOF mutation in human Slack channels^31,32^. Furthermore, increased susceptibility to glutamatergic excitotoxicity of Slack^−/−^ was also shown earlier^21^ as well as increased seizure susceptibility in *Drosophila*^43^ and mice^44^. The effects observed here are most likely due to the lack of Slack expression and not due to compensation by the related K^+^ channels Slick and BK or ionotropic glutamate receptors, as the respective transcript levels in both genotypes are similar 24 h after seizures (Supplementary Fig. 1). Interestingly, transcript levels of the neurotrophic factor BDNF were lower at that time point (Supplementary Fig. 1f), indicating again that Slack^−/−^ fail to upregulate neurotrophic signaling after detrimental excitatory events, as previously observed after glutamate-induced excitotoxicity^21^. Interestingly, elevated seizure susceptibility of Slack^−/−^ mice was previously demonstrated in an electroconvulsive model^44^. In that study, however, Slack^−/−^ mice showed increased post-ictal survival rates despite their increased sensitivity. This discrepancy might be due to different means of seizure induction. In contrast to KA injection, electric stimulation could preferentially activate cortical areas, leading to different excitatory and inhibitory excitation patterns. This fact, however, also indicates that Slack function may differentially affect the imbalanced neuronal activities in epileptogenesis depending on the involved cell types and brain areas as well as triggering non-genetic factors. Increased seizure severity in Slack^−/−^ is in line with an earlier hypothesis of Slack’s role as seizure terminator which was proposed shortly after the channel’s identification^45^. A great body of recent research, however, seems to contradict this notion, as GOF Slack variants appear to promote epilepsy development and severity in humans and mice^25,44,46,47^. Traditionally, K^+^ channels are seen as counter-epileptic agents^48,49^, but three possible pathomechanisms were discussed for how Slack overactivity causes seizures^29^. First, Slack channels increase AP frequencies by shortening voltage-gated Na^+^ channel (Na_V_) inactivation. Second, developmental alterations in synaptic connectivity led to the generation of hyperactive networks. Third, reduced excitability of inhibitory neurons disinhibits excitable networks. In support of the latter theory, altered inhibitory signaling in vivo and in vitro was demonstrated in three different knock-in (KI) mouse models of Slack GOF variants^44,46,47^. To reconcile this apparent epileptogenicity of Slack with our finding of increased seizure severity in Slack^−/−^, we must assume that too much as well as too little Slack activity possibly leads to detrimental neuronal activity in dependence on the involved brain regions. This raised the question of how the reduction or loss of Slack activity increases seizure incidence and severity.

Slack-mediated protection during KA-induced seizures was further explored by determining cell death after KA exposure in hippocampal brain slice cultures and primary dissociated hippocampal neurons. In agreement with our in vivo observations, both types of Slack^−/−^ hippocampal cell preparations were more susceptible to KA-induced damage than controls (Figs. 2 and 3). In slice cultures, cell death was predominant in CA3 (Supplementary Fig. 2), the hippocampal region with the highest expression of Slack^1,14^ and GluK^40^. Co-treatment of primary dissociated hippocampal neurons with PiTX exacerbated KA-induced cell death linking excessive neuronal activation by disinhibition to cell death (Fig. 3d). Overall, these experiments verified dissociated hippocampal neurons as a suitable model recapitulating important parts of the cellular events taking place during KA-induced seizures, which reportedly result in massive neuronal death^40^. Next, [Ca^2+^]_i_ was measured in response to increasing KA concentrations to assess the cell biological events leading to cell death and PI uptake in this system. Indeed, Slack limits Ca^2+^ influx at lower but not at high KA concentrations (Fig. 4c). This suggests a reduced threshold for Ca^2+^ entry into Slack^−/−^ neurons in agreement with the notion of K^+^ channels as inhibitory components, while maximal activation of Ca^2+^ influx remains unaffected. Surprisingly, reduction of [K^+^]_i_ in response to 1, 3, and 5 µM KA was much stronger in Slack^−/−^ (Fig. 4e, f). This finding indicates that exposing Slack^−/−^ neurons to KA (see below) activates an additional K^+^ conductance which, in turn, reduces [Ca^2+^]_i_ elevations (Fig. 4c). This data contrasts previous findings of decreased K^+^ efflux from Slack^−/−^ cerebellar granule cells after treatment with 300 µM NMDA^21^, but might be explained by high KA sensitivity throughout the CA1 and CA3 regions^40^ and relevant differences in cellular systems. Whole-cell current-clamp recordings provided further insights, how differences in Ca^2+^ and K^+^ handling observed in Slack^−/−^ hippocampal neurons translate into altered vulnerability to excitatory stimuli and ultimately more severe seizures in vivo. First, amplitudes of TTX-sensitive transient inward currents induced by depolarizing voltage steps were higher in Slack^−/−^ neurons. At the same time, steady-state currents at membrane voltages between −20 to +20 mV were inward-directed indicating the presence of increased I_NaP_ that were previously reported for *Drosophila* neurons deficient in the Slack analogue Slo2^43^ as well as in mammalian neurons^11,17^. Additionally, decreased I_NaP_ was reported in GABAergic cortical neurons of mice carrying a Slack GOF variant^47^, which is in line with our findings for Slack LOF neurons. Second, firing frequencies of AP trains induced by KA application or depolarizing current injection were significantly higher in Slack^−/−^ than in controls (Fig. 6b, f). RMP (Fig. 6g), AP amplitude and aHP amplitudes (Fig. 6i and Supplementary Data 1) were not different between genotypes. While our findings on AP amplitudes are similar to data from dorsal root ganglia and glutamatergic neurons of the basolateral amygdala^12,50^, those studies report slightly depolarized RMP. No altered RMP, however, was reported for GABAergic and glutamatergic cortical neurons carrying a Slack GOF variant^47^. These facts again underline that the impact of Slack channel function depends on the specific cellular system or tissue. Additionally, AP were elicited at more negative membrane voltages in Slack^−/−^ (Fig. 6h) together with increased maximal AP rise slopes (Fig. 6k), indicating that increased net inward currents found in Slack^−/−^ might influence discrete AP kinetics. Besides the raising phases of the AP, Slack^−/−^ also display a shorter aHP (Fig. 6n). These findings are in line with findings in other models: Slack^−/−^ dorsal root ganglion neurons show accelerated and deregulated firing patterns as well as increased AP rise slope and decreased AP threshold^12,33^. Also, Slack-deficient glutamatergic neurons from the basolateral amygdala respond with increased AP firing rates to current injection while Slack GOF neurons respond with slower firing^50^. The described reduction of the threshold depolarization necessary for eliciting AP might explain the increases in AP frequencies, Ca^2+^ influx and ultimately cell death as well as seizure severity observed in Slack^−/−^. Accordingly, the absence of the Slack channel in genetically and pharmacologically induced epilepsy models of *Drosophila* promoted seizure-like neuronal activity. It was thus concluded that the repolarizing K^+^ currents mediated by Slack serve as a protective brake against overexcitation^43^. Combined with our current data we think that during depolarization, a Slack-mediated sustained K^+^ outflow is involved in setting Na_V_ activation and thus AP threshold (Figs. 5c, 6n, and 6o). This subthreshold Slack current is probably activated by persistent sodium currents (Fig. 5d, e)^17^. In Slack^−/−^, however, this Na_V_ activation brake is removed to allow Na_V_ activation and AP initiation at slightly more negative membrane potentials. This relative increase in depolarizing Na^+^ currents would, in turn, allow increased AP frequencies to ultimately boost [Ca^2+^]_i_ and neuronal demise. Additionally, facilitated depolarization could also increase activation of voltage-gated K^+^ channels (K_V_), explaining the increased [K^+^]_i_ after stimulation with KA (Fig. 4f) and also faster aHP that provides faster repolarization for subsequent AP (Fig. 6f, n, o). Several members of the K_V_1 family, for example, are activated between −20 and −30 mV of membrane depolarization and could thereby affect AP frequencies^51^.

We identified the following limitations of our study: (1) This study exclusively used male mice for in vivo experiments. Our findings might therefore not be transferable to female subjects. (2) Slack^−/−^ animals carry a global Slack deletion. Hence, alterations in brain development^22^ and compensatory regulations of other channels cannot be excluded. (3) The hippocampal formation is known to be tightly linked to epileptogenesis^52^ and is heavily affected by seizures^53^. The KA-based model was reported to induce highly isomorphic seizures and hippocampal sclerosis-like damage in mice^40^. However, a direct link to Slack-related epilepsies that are characterized and studied on a cortical level and seizures frequently located in the frontal cortex^47,54^ is somewhat limited. (4) Impact of seizures was analyzed 24 h after KA injection on mRNA level. Whether posttranslational modifications and compensation on a protein level in response to loss of Slack affect long-term outcomes is not reported here. (5) Further work on additional brain regions, with differentiation between excitatory and inhibitory neurons combining, for instance, conditional Slack knockout models and analysis of seizure-induced subcellular protein expression might complete our findings. (6) Voltage-clamp recordings were conducted to observe altered Na^+^-dependent K^+^ currents. Due to the chosen experimental conditions, we cannot draw any conclusions about whether other conductances, e.g., voltage-gated Na^+^ of Ca^2+^ channels are changed in Slack^−/−^.

Taken together, our data imply that neuronal Slack activity needs to be tightly balanced, as excessive, and insufficient Slack function is detrimental depending on the cell types, tissues, and the factors triggering neuronal activity involved. In our study, increased excitability offers an explanation for severe seizures in a Slack LOF mutation^32^. It also urges caution when aiming for full Slack inhibition to treat GOF variant-induced epilepsies. It might be necessary to either develop partial Slack antagonists and/or to deliberate dosage, as too little Slack activity might well increase seizure susceptibility.

### Supplementary information

Supplementary Information

Description of Additional Supplementary Files

Supplementary Data 1

Reporting Summary

## Data Availability

This study includes no data deposited in external repositories. Source data underlying figures are provided in Supplementary Data 1. Further information and requests for source data should be directed to and will be fulfilled by the Lead Contact, Robert Lukowski (robert.lukowski@uni-tuebingen.de).
