# Supplementary material for: Slack K+ channels limit kainic acid-induced seizure severity in mice by modulating neuronal excitability and firing
Source: Commun Biol. 2023 Oct 11;6:1029. doi: 10.1038/s42003-023-05387-9 (PMC10567740; doi:10.1038/s42003-023-05387-9)
Supplement: Supplementary file 1 — Supplementary Information [file 42003_2023_5387_MOESM1_ESM.pdf]

Supplementary Information for the manuscript

Slack K+ channels limit kainic acid-induced seizure severity in mice by modulating neuronal excitability and firing

David Skrabak[1], Helmut Bischof[1], Thomas Pham[1], Peter Ruth[1], Rebekka Ehinger[1], Lucas Matt[1,§], Robert Lukowski[1,§]

[1.]Department of Pharmacology, Toxicology and Clinical Pharmacy, Institute of Pharmacy, University of Tübingen, Tübingen, Germany

[§] These authors contributed equally

Corresponding author: Robert Lukowski, email: robert.lukowski@uni-tuebingen.de

Key words: Na^+-activated K^+ channel, Slack, epilepsy, kainic acid

Supplementary Table 1: Oligonucleotides

	primer	sequence 5' to 3'
**genotyping**		
Slack	for	AGGGGCTGAGAGGGGTCTCG
	rev1	TGGGTAGGGCTGCCACAAGC
	rev2	GCCACAATCTGTTCCTTGGCA
**qRT-PCR**		
HPRT	for	CCTTCATGACATCTCGAGCA
	rev	CATTATGCCGAGGATTTGGA
Slack	for	CTGCTGTGCCTGGTCTTCA
	rev	AAGGAGGTCAGCAGGTTCAA
Slick	for	CTCGCGCTTTCAAAACTGGA
	rev	ACTCTTCCCGCAGCAAAAGG
BK	for	GACGCCTCTTCATGGTCTTC
	rev	TAGGAGCCCCCGTATTTCTT
GluK4	for	GCCTCATGAACTACCTTCGCA
	rev	GTTGGACCTCTGGCCTTTGC
GluK5	for	CCGTGTGGCTCTTCATGCTT
	rev	TGTACCACTCATAAGGGCTCAG
GluA1	for	GGTTGGCGAGGATGTAGTGG
	rev	TGGTGGTGGTGGACTGTGAA
GluA2	for	AGCACTCCTTAGCTTGATTGAGT
	rev	CCACTTCTTCTCCGCAGCAG
GluN1	for	AGGAAGATCATCTGGCCAGGA
	rev	GGGCTTGACATACACGAAGGG
GluN2A	for	GAGACCCCGCTACACACTC
	rev	TCAGCACGATCACCACAAGC
GluN2B	for	CGCCCAGATCCTCGATTTCA
	rev	ACTGGAAGAACATGGAGGACTCA
BDNF	for	GACGACATCACTGGCTGACA
	rev	GTCCGCGTCCTTATGGTTTT
TrkB	for	TTTCCGCCACCTTGACTTGT
	rev	TCCTGGAGAGTCTTGAGCCA
GFAP	for	TGCAAGAGACAGAGGAGTGGT
	rev	GTGCTTGGCTTGGCGGAG

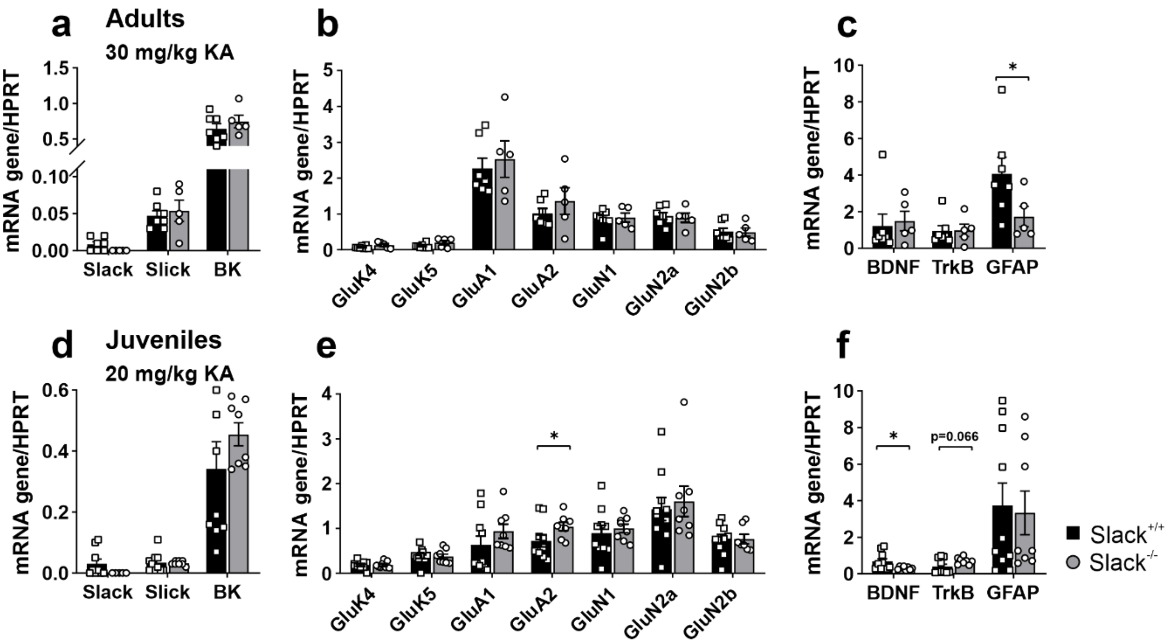

Supplementary Figure 1. mRNA expression levels of related genes are similar in Slack$^{+/+}$ and Slack$^{-/-}$ following KA-induces convulsions

a-c mRNA expression levels in 12-weeks-old adult male Slack$^{+/+}$ and Slack$^{-/-}$ mice tested 24 h after KA-induced seizures. No compensatory regulations of related K^+ channels **a**, glutamate-receptor subunits **b** or growth factors **c**, but significantly (unpaired t-test $p = 0.013$) decreased levels of *GFAP* **c** were found in Slack$^{-/-}$ compared to Slack$^{+/+}$.

d-f mRNA expression levels in 4-weeks-old juvenile mice tested 24 h after KA-induced seizures. No compensatory regulations of related K^+ channels **a** or glutamate-receptor subunits **b** were found in Slack$^{-/-}$. Interestingly Slack$^{-/-}$ mice respond to seizures with significantly (unpaired t-test $p = 0.047$) decreased *BDNF* transcription **f**.

Data represented as mean ± SEM, $p < 0.001$. For statistics also see Supplementary Data.

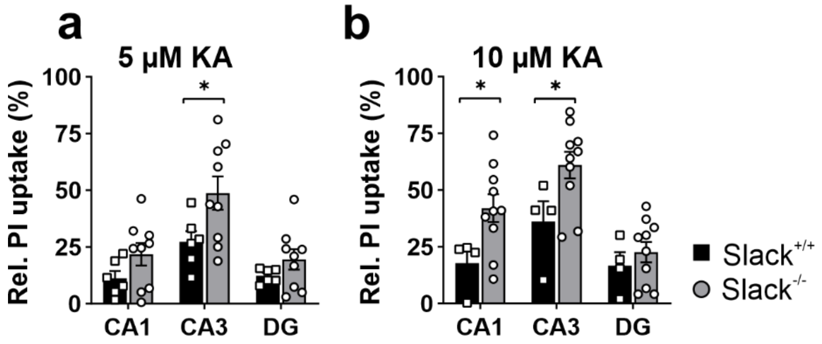

Supplementary Figure 2. Increased KA-induced cell death in Slack$^{-/-}$ organotypic hippocampal slice cultures due to predominant PI uptake in CA3 region

a Compared to Slack$^{+/+}$, Slack$^{-/-}$ slice cultures showed significantly increased PI uptake (normalized to maximum) in the hippocampal CA3 region after 24 h treatment with 5 µM KA (Slack$^{+/+}$ n = 6, Slack$^{-/-}$ n = 9, unpaired t-test p = 0.049).

b Compared to Slack$^{+/+}$ (n = 4), Slack$^{-/-}$ (n = 9) slice cultures show significantly increased PI uptake at CA1 (unpaired t-test p = 0.038) and CA3 (unpaired t-test p = 0.041) but not DG in response to 24 h treatment with 10 µM KA.

Data represented as mean ± SEM with $p < 0.01$. For statistics also see Supplementary Data.
