# Supplementary material for: Slack K+ channels limit kainic acid-induced seizure severity in mice by modulating neuronal excitability and firing
Source: Commun Biol. 2023 Oct 11;6:1029. doi: 10.1038/s42003-023-05387-9 (PMC10567740; doi:10.1038/s42003-023-05387-9)
Supplement: Supplementary file 2 — Description of Additional Supplementary Files [file 42003_2023_5387_MOESM2_ESM.pdf]

Description of Additional Supplementary Files

File Name: Supplementary Data 1
Description: Source data and statistics underlying all graphs in the manuscript.
