# Supplementary material for: Slack K+ channels limit kainic acid-induced seizure severity in mice by modulating neuronal excitability and firing
Source: Commun Biol. 2023 Oct 11;6:1029. doi: 10.1038/s42003-023-05387-9 (PMC10567740; doi:10.1038/s42003-023-05387-9)
Supplement: Supplementary file 4 — Reporting Summary [file 42003_2023_5387_MOESM4_ESM.pdf]

nature portfolio

COMMSBIO-23-0878

Corresponding author(s): Robert Lukowski, Tübingen, Germany

Last updated by author(s): Sep 13, 2023

Reporting Summary

Nature Portfolio wishes to improve the reproducibility of the work that we publish. This form provides structure for consistency and transparency in reporting. For further information on Nature Portfolio policies, see our Editorial Policies and the Editorial Policy Checklist.

Statistics

For all statistical analyses, confirm that the following items are present in the figure legend, table legend, main text, or Methods section.

n/a	Confirmed	
☐	☒	The exact sample size (*n*) for each experimental group/condition, given as a discrete number and unit of measurement
☐	☒	A statement on whether measurements were taken from distinct samples or whether the same sample was measured repeatedly
☐	☒	The statistical test(s) used AND whether they are one- or two-sided<br>*Only common tests should be described solely by name; describe more complex techniques in the Methods section.*
☐	☒	A description of all covariates tested
☐	☒	A description of any assumptions or corrections, such as tests of normality and adjustment for multiple comparisons
☐	☒	A full description of the statistical parameters including central tendency (e.g. means) or other basic estimates (e.g. regression coefficient) AND variation (e.g. standard deviation) or associated estimates of uncertainty (e.g. confidence intervals)
☐	☒	For null hypothesis testing, the test statistic (e.g. *F*, *t*, *r*) with confidence intervals, effect sizes, degrees of freedom and *P* value noted<br>*Give P values as exact values whenever suitable.*
☐	☒	For Bayesian analysis, information on the choice of priors and Markov chain Monte Carlo settings
☐	☒	For hierarchical and complex designs, identification of the appropriate level for tests and full reporting of outcomes
☐	☒	Estimates of effect sizes (e.g. Cohen's *d*, Pearson's *r*), indicating how they were calculated

Our web collection on statistics for biologists contains articles on many of the points above.

Software and code

Policy information about availability of computer code

Data collection	PatchMaster 2x91, VisiView
Data analysis	PatchMaster 2x91, Clampfit 10.7, GraphPad Prism 8, ImageJ

For manuscripts utilizing custom algorithms or software that are central to the research but not yet described in published literature, software must be made available to editors and reviewers. We strongly encourage code deposition in a community repository (e.g. GitHub). See the Nature Portfolio guidelines for submitting code & software for further information.

Data

Policy information about availability of data

All manuscripts must include a data availability statement. This statement should provide the following information, where applicable:

- Accession codes, unique identifiers, or web links for publicly available datasets
- A description of any restrictions on data availability
- For clinical datasets or third party data, please ensure that the statement adheres to our policy

Data are summarized in supplementary data tables as mean, SEM with statistical analysis. For raw data, please contact the corresponding author.

Human research participants

Policy information about studies involving human research participants and Sex and Gender in Research.

Reporting on sex and gender	not applicable
Population characteristics	not applicable
Recruitment	not applicable
Ethics oversight	not applicable

Note that full information on the approval of the study protocol must also be provided in the manuscript.

Field-specific reporting

Please select the one below that is the best fit for your research. If you are not sure, read the appropriate sections before making your selection.

☒ Life sciences ☐ Behavioural & social sciences ☐ Ecological, evolutionary & environmental sciences

For a reference copy of the document with all sections, see nature.com/documents/nr-reporting-summary-flat.pdf

Life sciences study design

All studies must disclose on these points even when the disclosure is negative.

Sample size	Sample sizes for in-vivo and in-vitro studies were determined a priori using the G*Power software suite to optimize for optimal statistical power and minimal animals needed. Prediction was based on previous studies that provide profound outlook on outcome and variances.
Data exclusions	Data sets were analyzed by Prism for outliers. Since no outliers were found in any published data, no data were excluded in this publication
Replication	Reproducibility was provided by repeating experiments using different lots of chemicals, preparations and conducting persons on a broad time frame. Consistency of data and variance between conditions are aproved for all experimental setups and data.
Randomization	Male animals of both genotypes were randomly allocated to in vivo experiments.
Blinding	All in-vivo testing was conducted by persons blinded to the animals' genotype. For in-vitro approaches genotype of tissue and dissociated neurons was not blinded. Analysis of all in-vitro data (PI uptake, Fura2, FRET and patch-clamp),however, were automated to avoid bias.

Reporting for specific materials, systems and methods

We require information from authors about some types of materials, experimental systems and methods used in many studies. Here, indicate whether each material, system or method listed is relevant to your study. If you are not sure if a list item applies to your research, read the appropriate section before selecting a response.

Materials & experimental systems

n/a	Involved in the study	
☐	☒	Antibodies
☒	☐	Eukaryotic cell lines
☒	☐	Palaeontology and archaeology
☐	☒	Animals and other organisms
☒	☐	Clinical data
☒	☐	Dual use research of concern

Methods

n/a	Involved in the study	
☒	☐	ChIP-seq
☒	☐	Flow cytometry
☒	☐	MRI-based neuroimaging

Antibodies

Antibodies used

KCNT1 primary antibody
Sigma Aldrich Chemie GmbH
Cat.#: SAB5200036
Clone #: S3-26 monoclonal
Lot #: 1004

MAP2 primary antibody
Manufacturer: Cell Signaling Tecnology
Cat.#: 8707
Clone #: D5G1
Lot #: 6

Alexa Fluor™ 555 secondary antibody
Manufacturer: Thermo Fisher Scientific
Cat.#: A21127
Clone #: polyclonal
Lot #: 2335727

Alexa Fluor™ 488 secondary antibody
Manufacturer: Thermo Fisher Scientific
Cat.#: A11034
Clone #: polyclonal
Lot #: 1771339

Validation

KCNT1 primary antibody
Species: mouse
Application: Western Blot, Immunihistochemistry
Reactivity: rat, humain, mouse
MW (kDa): ~140
Source/Isotype: IgG1
Immunogen: Fusion protein amino acids 1168-1237 of rat Slo2.2 (Slack); NP_068625
Databases: NCBI: NP_068625, UniProt: Q5JUK3
Citation:
Duyen H Pham et al., Human molecular genetics, 26(11), 2042-2052 (2017-03-24)

MAP2 primary antibody
Species: rabbit
Application: Western Blotting, Immunoprecipitation, Immunofluorescence (Frozen)
Reactivity: M, R
MW (kDa): 75, 82, 280
Source/Isotype: Rabbit IgG
Specificity / Sensitivity: MAP2 (D5G1) XP® Rabbit mAb recognizes endogenous levels of total MAP2 protein. Non-specific labeling of mouse pancreas, colon, small intestine, and liver may be observed by immunofluorescence.
Species Reactivity: Mouse, Rat
Source / Purification: Monoclonal antibody is produced by immunizing animals with a synthetic peptide corresponding to residues near the carboxy terminus of human MAP2 protein.
Citation:
Sanchez, C. et al. (2000) Prog. Neurobiol. 61, 133-168.
Berling, B. et al. (1994) Eur. J. Cell Biol. 64, 120-130.
Sanchez, C. et al. (2000) Eur. J. Cell Biol. 79, 252-260.

Database Links: UniProt ID: P11137, Entrez-Gene Id: 4133

Animals and other research organisms

Policy information about studies involving animals; ARRIVE guidelines recommended for reporting animal research, and Sex and Gender in Research

Laboratory animals

Species: Mus musculus
Strains:
1. Slack+/+: C57BL/6 or B6-Kcnt1+/+/RoLu
2. Slack-/-: Kcnt1tm1Ruth/RoLu

Wild animals

not applicable

Reporting on sex

In this study a total of 33 male mice were used in a Kainic acid-based model for acute epilepsy.
No in vivo experiments were performed on female animals, in line with previous studies to provide good comparability and to reduce statistical variance. Limitation of data based on exclusively male animals is discussed in the manuscript.
Organotypic and dissociated neuronal cultures used for in vitro experiments were generated from mouse pups of both sexes.

Field-collected samples

not applicable

Ethics oversight

The study protocols (PZ3/20 G, PZ 04/22 M, PZ 01/21 M) were approved by the Ethics Committee for Animal Experiments - Regierungspräsidium Tübingen in accordance with the German Animal Protection Act.

Note that full information on the approval of the study protocol must also be provided in the manuscript.
